# Authors’ Reply to: Challenges in Measuring What Matters to Patients With Diabetes. Comment on “Measurement Properties of Patient-Reported Outcome Measures for Diabetes: Systematic Review”

**DOI:** 10.2196/37957

**Published:** 2022-03-31

**Authors:** Yu Heng Kwan, Jie Kie Phang, Sungwon Yoon, Lian Leng Low

**Affiliations:** 1 Health Services and Systems Research Duke-NUS Medical School Singapore Singapore; 2 SingHealth Internal Medicine Residency Programme Singapore Health Services Singapore Singapore; 3 Department of Pharmacy National University of Singapore Singapore Singapore; 4 Centre for Population Health Research and Implementation SingHealth Regional Health System Singapore Singapore; 5 Population Health & Integrated Care Office (PHICO) Singapore General Hospital Singapore Singapore; 6 Department of Family Medicine and Continuing Care Singapore General Hospital Singapore Singapore; 7 Post-Acute and Continuing Care Outram Community Hospital Singapore Singapore; 8 SingHealth Duke-NUS Family Medicine Academic Clinical Program Singapore Singapore

**Keywords:** systematic review, measurement properties, patient-reported outcome measures, methodological quality, level of evidence, PROMs, patient reported outcome, diabetes

We would like to respond to the letter written by Rutters et al [1] with regard to our paper, “Measurement Properties of Patient-Reported Outcome Measures for Diabetes: Systematic Review” [2]. We noted the concerns from Rutters et al [1], but we would like to offer some explanations.

First, the selection criteria of our systematic review were restricted to patient-reported outcome measures (PROMs) that are tested in patients with type 2 diabetes mellitus (T2DM) only. The study on the development and validation of the National Diabetes Register Survey included patients with other forms of diabetes (ie, type 1 diabetes) [3], and, therefore, was excluded from our analysis. We focused on T2DM since existing evidence has demonstrated that patients’ behaviors influencing disease management differ by different diabetes subtypes [4,5]. Therefore, the PROMs used to guide interventions and patient care may be different and should be reviewed separately. Another consideration was related to our concerns that combining all validation studies of PROMs in different forms of diabetes would reduce the readability of the paper due to the large number of studies available.

Second, due to the large number of PROMs included in the review, we decided to analyze the measurement properties of the PROMs on a per-PROM basis instead to maintain the readability of the paper. We also agree with Rutters et al [1] that many health-related quality of life (HRQOL) PROM subscales do not measure HRQOL but actually measure overall quality of life, and that characteristics of the individual or environment should be considered patient-reported experience measures. This is further complicated by the issue of problematic definitions of HRQOL in the literature [6]; thus, further study detailing the different constructs measured by subscales of PROMs is warranted.

We are grateful that the authors have taken the effort to provide constructive comments on our paper. The issues brought up by Rutters et al [1] echoed the need to have consensus between clinicians and psychometrists to measure what is relevant to patients. The content of the existing PROMs is indeed heterogeneous, and there are too many PROMs that have questionable validity. We agree that more awareness is needed, including developing and implementing core outcome sets for patients with diabetes.

In conclusion, there is a need for a systematic review to summarize all available PROMs for patients with diabetes with emphasis on the constructs being measured, as well as a comprehensive evidence synthesis of the measurement properties of all subscales of PROMs (which was not the focus of our systematic review). Clinicians and researchers should work with patients with diabetes to develop a core outcome measurement set for use in diabetes care and research.

## Abbreviations

HRQOL: health-related quality of life
PROM: patient-reported outcome measure
T2DM: type 2 diabetes mellitus
